# Racial disparities in pre-operative pain, function and disease activity for patients with rheumatoid arthritis undergoing Total knee or Total hip Arthroplasty: a New York based study

**DOI:** 10.1186/s41927-020-0117-0

**Published:** 2020-02-29

**Authors:** J. Hirsch, B. Mehta, J. Finik, I. Navarro-Millan, C. Brantner, S. Mirza, M. Figgie, M. Parks, L. Russell, D. Orange, S. Goodman

**Affiliations:** 1 Mount Sinai St. Luke’s-West, New York, NY USA; 2 Hospital for Special Surgery, 535 E 70th Street, New York, NY 10021 USA; 3 Weill Cornell Medicine, New York, NY USA; 4 The Rockefeller University, New York, NY USA

**Keywords:** Racial disparity, Minority groups, Rheumatoid arthritis (RA), Arthroplasty, Total knee arthroplasty (TKA), Total hip arthroplasty (THA), Pre-operative

## Abstract

**Background:**

Black and Hispanic patients with osteoarthritis have more pain and worse function than Whites at the time of arthroplasty. Whether this is true for patients with rheumatoid arthritis (RA) is unknown.

**Methods:**

This cross-sectional study used data on RA patients acquired between October 2013 and November 2018 prior to elective total knee (TKA) or hip arthroplasty (THA). Pain, function, and disease activity were assessed using the visual analogue scale (VAS), the Multidimensional Health Assessment Questionnaire (MDHAQ), and the Disease Activity Score (DAS28-ESR). We linked the cases to census tracts using geocoding to determine the community poverty level. Race, education, income, insurance and medications were collected via self-report. Using multivariable linear and logistic models we examined whether minority status predicted pain, function and RA disease activity at the time of arthroplasty.

**Results:**

Thirty seven (23%) of the 164 patients were Black or Hispanic (minorities). The MDHAQ and DAS28-ESR were not significantly worse while VAS pain score was significantly worse in minority patients (*p* = 0.03). There was no significant difference in education between the groups. Insurance varied significantly; 29% of minority patients had Medicaid vs. 0% of Whites (*p* < 0.0001). In the multivariable analyses minority status was not significantly associated with DAS28-ESR [*p* = 0.66], MDHAQ [*p* = 0.26], or VAS pain [*p* = 0.18].

**Conclusions:**

For Black and/or Hispanic patients with RA undergoing THA or TKA at a high-volume specialty hospital, unlike Black or Hispanic patients with osteoarthritis (OA), there was no association with worse pain, function, or RA disease activity at the time of elective arthroplasty.

## Background

Socioeconomic factors including race, poverty and educational attainment may affect multiple health outcomes, including arthroplasty utilization, and pain and function after arthroplasty [1]. Interactions between social factors may be complex. For instance, race and poverty are associated with worse arthroplasty outcomes, but education may mitigate the effect of poverty within poor communities [2]. In addition, worse arthroplasty outcomes associated with Black race are not seen for Black patients residing in communities with less than 10% poverty [3]. Black and Hispanic patients with OA undergoing total hip (THA) and total knee arthroplasty (TKA), when compared to White patients, present with more pain and worse function at baseline [3] and seek arthroplasty with more severe disease [4]. As baseline status is strongly linked to outcomes [5], late presentation may have long-term negative consequences.

For patients with RA, therapy utilization, disease activity, and health-related quality of life are also linked to race, socioeconomic status, and educational attainment [6, 7]. Similarly, Black and Hispanic patients with RA who received ongoing care for their disease in community-based practices have previously been shown to have worse pain and function and higher disease activity scores compared to White patients [8].

It is not known if Black or Hispanic patients with RA undergoing arthroplasty have more pain, worse function, and higher disease activity at the time of THA and TKA compared to White patients with RA. The objective of this study was to assess the pain, function, and disease activity status of Black and Hispanic patients with RA at the time of presentation for elective THA and TKA. We hypothesized that patients with RA who are Black or Hispanic, similar to Black or Hispanic patients with osteoarthritis (OA), would have worse pain [9] and function [10] at the time of THA and TKA, and that higher educational attainment would mitigate the effect of race.

## Methods

### Study design and setting

This is a cross-sectional cohort study using data acquired prospectively from October 2013 through November 2018. Black, White, and Hispanic patients with RA undergoing elective THA and TKA at a single, high-volume tertiary care center for musculoskeletal diseases were recruited prior to surgery and underwent a comprehensive evaluation, including patient- and physician-reported measures and laboratory evaluations performed pre-operatively. Confirmed cases were included in the study after informed consent was obtained.

### Participants

Patients undergoing elective THA or TKA who were ≥ 18 were screened via electronic medical record to identify patients with RA. Patients were included if they met American College of Rheumatology/European League Against Rheumatism (ACR/EULAR) 2010 or 1987 criteria [11] or if the diagnosis was confirmed by the principal investigator (SG). We excluded patients with crystalline arthropathy or other rheumatic diseases including systemic lupus erythematosus, ankylosing spondylitis, and psoriatic arthritis, those who were unable to understand or read English, or those deemed unreliable to follow the study protocol.

### Exposure and outcomes

The exposure was minority status (self identified as Black or Hispanic) among patients with RA undergoing arthroplasty. The outcomes of interest in our study were pre-operative function [the Multidimensional Health Assessment Questionnaire (MDHAQ)], pain [the Visual Analogue Scale (VAS)], and disease activity [the Disease Activity Score (DAS28)]. The exposure and outcomes were measured pre-operatively.

### Data collection

Baseline data collected included age, sex, education level, and medications. We grouped patients into minority and non-minority cohorts based on self-identification. The minority group included Hispanic, Black or African American, and mixed race patients; the non-minority group were White patients. Poverty at the community level was determined using census tract data, by linking individual patients’ addresses via geocoding to their respective census tracts. We obtained census tract-level socioeconomic variables from the American Community Survey/United States Census using the Arc Geographic Information Systems. We used 20% of the population living below poverty as the point below which health impacts are reported [12, 13]. The census tract variable of interest was “percentage of families and people whose income in the last 12 months is below the poverty level - All families”.

We assessed patients using patient and provider global assessments as well as tender and swollen joint counts. Inflammatory marker measurement including erythrocyte sedimentation rate (ESR) and C-reactive protein (CRP), rheumatoid factor (RF) and anti-citrullinated protein antibody (ACPA) were obtained within two weeks prior to surgery or on the day of surgery, prior to the surgical incision.

Medication use was per standard of care and was recorded. Biologic usage at the time of surgery was captured and analyzed as a binary categorical variable. Patients who discontinued their biologics for the surgery were considered to be taking biologics. Insurance status included Medicaid, Medicare, or commercial. Medicaid is a public assistance program providing insurance to those in need based on income, and Medicare is an age-based federal insurance program provided to people above age 65 as well as those with disabilities. Medicaid insurance status was used as a proxy for income at the individual level, given the inaccuracies of self-reported income assessments [14]. Those presenting with two types of insurance were categorized into one of the existing insurance status categories according to the following priority: Medicaid followed by Medicare and finally commercial insurances. Patients were marked as having one type of insurance in these cases. For example, a patient who had both Medicare and commercial insurance was marked as having Medicare. Education was recorded as a binary categorical variable with those achieving some college or greater differentiated from those who achieved high school or less.

Function was assessed using the validated MDHAQ, which provides patients with specific questions (example: ability to get in and out of bed) in an effort to assess their overall functional status. The score is comprised of 10 activities of daily living which are scored from 0 to 3 with a score of 0 implying no difficulty performing the activity and 3 implying an inability to perform the activity. The score total is 0–30; a higher number indicates a worse functional status [15]. Pain was measured using the visual analogue scale (VAS) pain score, which is a user friendly scoring system where patients indicate their level of pain on a spectrum from 1 to 10, with 10 being unbearable pain [16]. Both MDHAQ and VAS were recorded as continuous variables. The DAS-28 [17] is a composite measure of disease activity that includes swollen and tender joint counts performed by the physician, plus the ESR and the patient’s global assessment of their health (PGA) [18]. A higher DAS-28 score implies a more active disease state. DAS28-ESR was chosen as the primary outcome of interest and as the measure of disease activity; it was binarized to patients with moderate or high disease activity (DAS28 ≥ 3.2) and those with low disease activity or remission of disease (DAS28 < 3.2).

### Statistical analysis

The distribution of continuous baseline patient characteristics was assessed for normality using the Shapiro-Wilk test. Normally distributed values were summarized as mean ± SD and compared using t-tests. If non-normally distributed, values were reported as median [25th percentile, 75th percentile] and compared using the Wilcoxon rank-sum test. Categorical variables were summarized as frequency and percent and compared across minority status using Fisher’s exact tests. Student t-test or the Wilcoxon rank-sum test were used, as appropriate, to compare continuous variables across minority status.

Univariate and multivariable linear regressions were performed to assess the impact of minority status on preoperative pain (VAS score), and function (MDHAQ), while univariate and multivariable logistic regressions were performed to assess odds of moderate/severe RA disease activity (DAS28-ESR). Results of linear models are summarized with slope(β) ± SE, while logistic models are summarized with odds ratios (OR) and 95% confidence intervals (CI). Mean and Standard Deviation (SD) were used for values that were normally distributed and median and Interquartile Range (IQR) were used for values that were not normally distributed. The following variables were forced into all multivariable models as they have been recognized as predictors of total joint replacement outcomes in previous studies and are relevant to the research interest: education, insurance status, age, sex, and biologics [19].

### Missing data

For missing data, we retained all observations that recorded race and insurance status in the univariate analyses. In the multivariable model, the maximum number of patients that had data for each variable included in the model were retained i.e. minority status, education, insurance type, age, sex, use of biologics and MDHAQ score were all required in order to be included in the multivariable model for odds of moderate/high DAS-28 ESR (Table 3). We excluded patients with missing values for race and insurance status. One White patient opted not to disclose gender but was included in the analysis. All analyses were performed using SAS version 9.4. A two-tailed *p* < 0.05 was considered statistically significant.

This study is reported per the Strengthening the Reporting of Observational Studies in Epidemiology (STROBE) checklist for cohort studies. This study was approved by the hospital institutional review board and all included patients provided informed consent.

## Results

Our cohort included 164 patients with rheumatoid arthritis undergoing elective THA or TKA, of which 37 (22.6%) were minorities defined as either Black or Hispanic, and 127 (77.4%) were White patients (Table 1). The mean age was 62.5, the majority were women (87%), and most achieved an education level of at least some college (88%). There was a trend towards younger age for minority patients, median (IQR): 56.8 (51.0–68.8), when compared to White patients, median (IQR): 64.0 (55.0–71.5), although this difference was not significant. When the patients were linked to census tracts via geocoding 5 (3%) of the cohort (*n* = 164) lived in census tracts with 20% or more of the population living below poverty level, 3 (60%) were in the minority group (2 Black, 1 Hispanic), and 2 (40%) were White.

**Table 1:** Demographics, overall and by minority status collected pre-operatively

Variable	Overall (*N* = 164)	Minority^a^ (*N* = 37)	White (*N* = 127)	*p*-value
Age, years, median [IQR]	62.5 [54.7, 71.2]	56.8 [51.0, 68.8]	64.0 [55.1, 71.5]	0.063
Sex, male	22 (13.41%)	3 (8.11%)	19 (15.08%)	0.41
BMI > 30	**66 (40.24%)**	**26 (70.27%)**	**40 (31.50%)**	**< 0.0001**
Ever smoker, yes	87 (53.05%)	18 (48.65%)	69 (54.33%)	0.55
Education, Graduated HS & -	18 (11.76%)	4 (12.90%)	14 (11.48%)	0.76
Some college & +	135 (88.24%)	27 (87.10%)	108 (88.52%)	
Insurance, N/A	23 (14.11%)	6 (16.22%)	17 (13.49%)	
Commercial	54 (33.13%)	9 (29.03%)	45 (41.28%)	**< 0.001**
Medicare	77 (47.24%)	13 (41.94%)	64 (58.72%)	
Medicaid	9 (5.52%)	9 (29.03%)	0 (0.0%)	
Biologics, Yes	90 (55.56%)	23 (62.16%)	67 (53.60%)	0.45 **0.029**
VAS Pain Score, median [IQR]	**6.0 [4.0, 8.0]**	**7.5 [4.0, 8.0]**	**6.0 [4.0, 8.0]**	
MDHAQ Score, mean ± SD	11.8 ± 5.3	12.3 ± 5.1	11.6 ± 5.3	0.53
DAS28-ESR, mean ± SD	3.8 ± 1.3	4.1 ± 1.3	3.8 ± 1.2	0.18

^a^Minority status: Black or African American, Hispanic or Mixed

Significant values are bolded. Percentages in parenthesis unless otherwise indicated

*IQR* Interquartile Range, *VAS* Visual Analogue Scale, *MDHAQ* Multidimensional Health Assessment Questionnaire, *DAS28-ESR* Disease Activity Score28-Erythrocyte Sedimentation Rate, *HS* High School

Most minority patients (87%) and White patients (88.5%) achieved an education level of at least some college, with no significant difference between the groups. The majority of the cohort was on biologics at the time of arthroplasty (56%); although not significant, a greater percentage of minority patients (62%) were on biologics than White patients (54%, *p* = 0.45). Insurance type was significantly different between minority and White groups (*p* < 0.001): 29% of minority group vs. 42% of White patients had commercial insurance, 42% of minority group vs. 59% in White group had Medicare, 29% of minority group vs. 0% of White patients had Medicaid. The median VAS pain score for the overall cohort was 6.0 (IQR; 4.0, 8.0), and was significantly higher among minorities (7.5 vs 6.0, *p* = 0.03). The mean MDHAQ score for the cohort was 11.8 (SD 5.3) and the mean DAS28-ESR score for the cohort was 3.8 (SD 1.), with no significant difference between groups (*p* = 0.53 and 0.18, respectively).

In the univariate analyses to determine predictors of RA disease activity (Table 2**)** education level and sex were significant predictors of RA disease activity. In our multivariable model (Table 3), MDHAQ score was a significant predictor of moderate/severe RA disease activity [OR = 1.09; 95% CI (1.0, 1.17), *p* = 0.04]. Although higher education level [OR = 9.57; 95% CI (2.2, 42.2), *p* = 0.003] was a statistically significant predictor of moderate/high RA disease activity, the difference in education between groups was small. Only 18 (11.8%) patients had an educational level of high school or less across both minority and White groups and the wide confidence intervals for education indicate that this estimate had limited precision.

**Table 2:** Univariate analysis to determine the risk of high RA disease activity (DAS28-ESR,Logistic Regressions)

DAS28-ESR	Odds ratio (95% CI)	*p*-value
Minority (Yes)^a^	1.16 (0.54, 2.49)	0.70
Education (Some college +)	**4.48 (1.60, 12.57)**	**0.004**
Medicare^b^	1.23 (0.62, 2.42)	0.55
Medicaid^b^	1.69 (0.39, 7.40)	0.49
Age	0.99 (0.96, 1.02)	0.38
Sex (Female)	**3.25 (1.34, 7.86)**	**0.009**
Biologics (Yes)	1.02 (0.54, 1.90)	0.97
MDHAQ Score	**1.11 (1.04, 1.19)**	**0.003**

^a^Minority status: Black or African American, Hispanic or Mixed

^b^Ref commercial

Significant values are bolded

*DAS28-ESR* Disease Activity Score28-Erythrocyte Sedimentation Rate, *β* Beta Coefficient

*SE* Standard Error, *MDHAQ* Multidimensional Health Assessment Questionnaire, *CI* Confidence Interval

**Table 3:** Multivariable analysis to determine the risk of RA disease activity (DAS28-ESR, Generalized Logistic Model)

Variable	Odds ratio (95% CI)	*p*-value
Minority^a^ (Yes)	1.29 (0.41, 4.05)	0.66
Education (Some college +)	**9.57 (2.17, 42.21)**	**0.003**
Medicaid^b^	4.30 (0.28, 66.69)	0.30
Medicare^b^	1.48 (0.49, 4.45)	0.48
Age	0.97 (0.92, 1.02)	0.22
Sex (Female)	2.35 (0.74, 7.49)	0.15
Biologics (Yes)	0.64 (0.27, 1.48)	0.30
MDHAQ Score	**1.09 (1.00, 1.17)**	**0.044**

^a^Minority status: Black or African American, Hispanic or Mixed

^b^Ref: Commercial

Significant values are bolded

*DAS28-ESR* Disease Activity Score28-Erythrocyte Sedimentation Rate, *β* Beta Coefficient, *SE* Standard Error, *MDHAQ* Multidimensional Health Assessment Questionnaire, *CI* Confidence Interval

In the univariate model to determine predictors of VAS pain at the time of presentation for arthroplasty, only MDHAQ (β 0.18 ± 0.04, *p* < .0001) was a significant predictor of worse pain at the time of arthroplasty (Table 4). In the multivariable model, after forcing variables of interest into the model, only MDHAQ remained a significant predictor of worse pain scores (β 0.17 +/− 0.05, *p* = 0.0002) (Table 5).

**Table 4:** Univariate analysis to determine the risk of pain (VAS Pain Score, Simple Linear Regression)

Variable	β ± SE	*p*-value
Minority^a^ (Yes)	1.01 ± 0.54	0.06
Education (Some college +)	0.66 ± 0.68	0.33
Medicare^b^	0.46 ± 0.51	0.36
Medicaid^b^	−0.13 ± 1.17	0.91
Age	−0.01 ± 0.19	0.70
Sex (Female)	0.62 ± 0.65	0.34
Biologics (Yes)	0.56 ± 0.45	0.21
MDHAQ Score	**0.18 ± 0.04**	**< 0.0001**

^a^Minority status: Black or African American, Hispanic or Mixed

^b^Ref commercial; see below for minority status definition

Significant values are bolded

*VAS* Visual Analogue Scale, *β* Beta Coefficient, *SE* Standard Error, *MDHAQ* Multidimensional Health Assessment Questionnaire

**Table 5:** Multivariable analysis to determine risk of pain (VAS Pain Score, Generalized Linear Model)

VAS Pain Score	β ± SE	*p*-value
Minority^a^ (Yes)	0.87 ± 0.66	0.18
Education (Some college +)	0.12 ± 0.77	0.87
Medicaid^b^	−0.13 ± 1.37	0.93
Medicare^b^	0.22 ± 0.67	0.75
Age	0.02 ± 0.03	0.44
Sex (Female)	0.16 ± 0.70	0.82
Biologics (Yes)	0.81 ± 0.50	0.11
MDHAQ Score	**0.17 ± 0.05**	**0.0002**

^a^Minority status: Black or African American, Hispanic or Mixed

^b^Ref: Commercial

Significant values are bolded

*VAS* Visual Analogue Scale, *β* Beta Coefficient, *SE* Standard Error, *MDHAQ* Multidimensional Health Assessment Questionnaire

In our univariate model to explore predictors of MDHAQ at the time of arthroplasty, we found no statistically significant predictive factors (Table 6). In our multivariable model after forcing in factors of interest, only age was a predictor of MDHAQ at the time of arthroplasty(β − 0.17 +/− 0.06, *p* = 0.004) (Table 7). Neither minority status nor Medicaid insurance were significant in any of our models to predict the status of our patients with RA at the time they seek arthroplasty.

**Table 6:** Univariate analysis to determine the risk of poor function y (MDHAQ Score,Simple Linear Regressions)

MDHAQ Score	β ± SE	*p*-value
Minority^a^ (Yes)	0.66 ± 1.03	0.53
Education (Some college +)	− 0.19 ± 1.27	0.88
Medicare^b^	0.12 ± 0.97	0.90
Medicaid^b^	0.71 ± 2.15	0.74
Age	−0.06 ± 0.04	0.065
Sex (Female)	1.28 ± 1.24	0.30
Biologics (Yes)	0.86 ± 0.84	0.31

^a^Minority status: Black or African American, Hispanic or Mixed

^b^Ref commercial; see below for minority status definition

Significant values are bolded

*MDHAQ* Multidimensional Health Assessment Questionnaire, *β* Beta Coefficient, *SE* Standard Error

**Table 7:** Multivariable analysis to determine risk of poor function (MDHAQ Score, Generalized Linear Model)

MDHAQ Score	β ± SE	*p*-value
Minority^a^ (Yes)	1.44 ± 1.27	0.26
Education (Some college +)	−0.24 ± 1.49	0.87
Medicaid^b^	−2.60 ± 2.66	0.33
Medicare^b^	2.31 ± 1.28	0.07
Age	**−0.17 ± 0.06**	**0.004**
Sex (Female)	0.26 ± 1.37	0.85
Biologics (Yes)	0.13 ± 0.97	0.90

^a^Minority status: Black or African American, Hispanic or Mixed

^b^Ref: Commercial

Significant values are bolded

*MDHAQ* Multidimensional Health Assessment Questionnaire, *β* Beta Coefficient, *SE* Standard Error

## Discussion

In this study we assess whether patients with RA who are Black or Hispanic have worse pain, function and disease activity at the time of arthroplasty and find no difference when compared to White patients with RA, unlike the experience of Black patients with osteoarthritis (OA) who present for arthroplasty with worse pain and function [20]. For Black and Hispanic patients with RA undergoing arthroplasty at a high-volume surgical hospital with high levels of education, who live in communities with little poverty, there is no difference in baseline pain or function compared to White patients. Similarly, disease activity and biologics use were no different between the groups.

Socioeconomic factors such as poverty and lower educational attainment are disproportionately seen among Black and Hispanic patients who are more likely to live in poor communities where health outcomes are poorer, so the contributions of “place vs. race” can be hard to discern [4]. Using geocoding and linking our cohort to individual census tracts, we demonstrated that there was little difference in this study in the proportion of residence in a census tract below the poverty line across minority status. Although Medicaid insurance, a reliable surrogate for individual level poverty, was significantly different between the groups in this cohort, educational levels and community resources were equivalent, and all received care in the same high-volume hospital. In this cohort, race and ethnicity are not associated with worse disease status at the time of arthroplasty.

Median household income is used to determine gradients in community poverty and offer a different perspective than using individual income [21]. In this study, while there was significantly more poverty at the individual level (Medicaid insurance) for minority patients, few in the cohort lived in poor communities and there was little difference in community level poverty between the groups.

Biologic disease modifying antirheumatic drugs (DMARDs) may be underutilized which may contribute to poorer disease status in Black patients with RA who have Medicare [22] and Medicaid [23] insurance. Black patients with the California Medicaid program (Medical), seen in outpatient and inpatient settings are reported to underutilize biologics when compared to White patients, and utilize DMARDs 6–11% less than other racial groups [24]. We demonstrate that in minority patients with long standing RA seen prior to arthroplasty, some of whom are insured by Medicaid, the utilization of biologic DMARDs is no different compared to White patients with RA with 56% of both groups using biologics. RA patients undergoing arthroplasty may represent a cohort of patients who are selecting more aggressive care overall, including biologics, as compared to other RA patients. Alternatively, the similarities in education and community poverty level may be mitigating the disparities described previously in utilization of therapies for patients with RA.

Several limitations regarding this study should be noted. While minority status was not found to be a significant predictor of any RA disease outcomes, this may be due to the characteristics of the present cohort. The cohort consisted of majority White females with some college education or higher, and we have previously demonstrated that education can mitigate the effects of poverty on arthroplasty outcomes, but in this study, both white and minority patients had similar education, limiting the potential for introducing bias [2]. As most patients receive care in community hospitals where a broader range of education and individual level income exists our results may not be generalizable. The study cohort is limited to patients who present for arthroplasty at a tertiary care specialty hospital in New York City, another limitation in generalizability. Racial or ethnic differences in pain perception or impact could bias these results; however, little difference in pain perception has been found between Blacks, Whites, or Hispanics [25]. Finally, as in any observational study, our results may be affected by unmeasured confounders.

Future studies would benefit from examination of this cohort of patients in the post-operative period where THA and TKA outcomes would be studied independently. Pre-operative factors such as radiographic severity, pain perception and catastrophizing as well as hip disability and osteoarthritis outcome score/knee injury and osteoarthritis outcome score (HOOS/KOOS) pain and function should be examined to assess the impact on the post-operative outcomes and post procedure complications.

## Conclusion

In summary, in a cohort of highly educated patients with RA receiving care at a high volume academic surgical hospital, residing in communities with low levels of poverty despite significant poverty at the individual level, we find that Black and Hispanic patients with RA do not have worse pain, function, or disease activity at the time of arthroplasty. There was no difference in biologic usage across minority status. Our findings lend further support to the concept of “place, not race” in the study of health care disparities.

## Data Availability

The datasets used and/or analyzed during the current study are available upon reasonable request.

## Abbreviations

ACPA: Anti-Citrullinated Protein Antibody
ACR/EULAR: American College of Rheumatology/European League Against Rheumatism
CRP: C-Reactive Protein
DAS28-ESR: Disease Activity Score-Erythrocyte Sedimentation Rate
DMARD: Disease Modifying Antirheumatic Drugs
ESR: Erythrocyte Sedimentation Rate
IQR: Interquartile Range
MDHAQ: Multidimensional Health Assessment Questionnaire
Medical: California Medicaid Program
OA: Osteoarthritis
OR: Odds Ratio
PGA: Patient’s Global Assessment
RA: Rheumatoid Arthritis
SAS: Statistical Analysis System
SD: Standard Deviation
STROBE: Strengthening the Reporting of Observational Studies in Epidemiology
THA: Total Hip Arthroplasty
TKA: Total Knee Arthroplasty
VAS: Visual Analogue Scale
β: Beta Coefficient of Slope
